# MiRNome variations in milk fractions during feed restrictions of different intensities in dairy cows

**DOI:** 10.1186/s12864-023-09769-5

**Published:** 2023-11-13

**Authors:** A. Leduc, S. Le Guillou, D. Laloë, L. Herve, J. Laubier, P. Poton, Y. Faulconnier, J. Pires, M. Gele, P. Martin, C. Leroux, M. Boutinaud, F. Le Provost

**Affiliations:** 1 Université Paris-Saclay, INRAE, AgroParisTech, GABI, Jouy-en-Josas, 78350 France; 2 PEGASE, INRAE, Institut Agro, 35590 Saint Gilles, France; 3 Institut de L’Elevage, 75012 Paris, France; 4 INRAE, Université Clermont Auvergne, VetagroSup, UMRH, Saint-Genès-Champanelle, 63122 France

**Keywords:** Dairy cows, Milk fractions, microRNA, miRNome, Feed restriction

## Abstract

**Background:**

In dairy cows, diet is one factor that can affect their milk production and composition. However, the effect of feed restriction on milk miRNome has not yet been described. Indeed, milk is the body fluid with the highest RNA concentration, which includes numerous microRNA. Its presence in the four different milk fractions, whole milk, fat globules, mammary epithelial cells and extracellular vesicles, is still poorly documented. This study aimed to describe the effects of different feed restrictions on the miRNome composition of different milk fractions.

**Results:**

Two feed restrictions were applied to lactating dairy cows, one of high intensity and one of moderate intensity. 2,896 mature microRNA were identified in the different milk fractions studied, including 1,493 that were already known in the bovine species. Among the 1,096 microRNA that were sufficiently abundant to be informative, the abundance of 1,027 of them varied between fractions: 36 of those were exclusive to one milk fraction. Feed restriction affected the abundance of 155 microRNA, with whole milk and milk extracellular vesicles being the most affected, whereas milk fat globules and exfoliated mammary epithelial cells were little or not affected at all. The high intensity feed restriction led to more microRNA variations in milk than moderate restriction. The target prediction of known microRNA that varied under feed restriction suggested the modification of some key pathways for lactation related to milk fat and protein metabolisms, cell cycle, and stress responses.

**Conclusions:**

This study highlighted that the miRNome of each milk fraction is specific, with mostly the same microRNA composition but with variations in abundance between fractions. These specific miRNomes were affected differently by feed restrictions, the intensity of which appeared to be a major factor modulating milk miRNomes. These findings offer opportunities for future research on the use of milk miRNA as biomarkers of energy status in dairy cows, which is affected by feed restrictions.

**Supplementary Information:**

The online version contains supplementary material available at 10.1186/s12864-023-09769-5.

## Background

Milk is a unique secretory product whose rich composition in nutrients and biological components, including microRNA (miRNA), is crucial to the development of neonates. Milk composition is affected by numerous factors such as genetics, environment, health status, lactation stage and nutrition [1–5]. For example, undernutrition in dairy cows can rapidly induce a negative energy balance that affects milk production and composition, and hence economic outcomes, and might also affect health, notably through an increased risk of ketosis [5]. Numerous studies on negative energy balance have used feed restriction experiments as a model; their effects greatly depend on their duration, intensity and the lactation stage at which they took place [5].

MiRNA are small single-stranded RNA (18–25 nucleotides) involved in the post transcriptional regulation of gene expression. Their base-pairing with mRNA inhibits their translation or induces their degradation [6]. As such, they are involved in the regulation of most biological processes. Milk is the body fluid that contains the highest concentration and variety of miRNA [7], which are present in different milk fractions such as milk fat, whey and cells with different profiles [8] and can also been found in extracellular vesicles (EV) which are fairly abundant in milk [9]. Recent reports have suggested that most milk miRNA is encapsulated within small EV [10]. Moreover, mammary epithelial cells (MEC) exfoliated and purified from milk are known to be a relevant source of mammary transcripts [11] and could thus be an interesting source of miRNA [8]. The milk composition in miRNA, *i.e.* the milk miRNome, has been described as being well conserved across species, and the top 10 most enriched miRNA sequences in milk appear to be quite similar in different milk fractions [12].

In dairy cows, the milk miRNome has been shown to vary within different breeds [4] or in a context of mastitis [13, 14], but has still not been studied during feed restriction. However, mammary gland miRNome is impacted by food deprivation in goats [15] and recently, Billa et al*.* [16] also observed mammary gland miRNome variations during feed restriction in dairy cows. It is therefore very likely that the bovine milk miRNome would also be modified by feed restriction. In human health, miRNAs are currently being studied extensively for their use as biomarkers of various pathologies such as cancer, epilepsy, sepsis, Alzheimer’s disease or cardiovascular diseases [17]. Milk miRNome modifications may therefore reflect mammary gland metabolism or even global metabolism adaptations to stresses such as the negative energy balance induced by feed restriction. The use of microRNA as biomarkers of these stresses should thus be considered.

The aim of the present study was therefore to describe the effects of feed restrictions of different intensities on the miRNome composition of different milk fractions in dairy cows in order to identify miRNA that might characterize this nutritional stress. Two feed restriction trials were applied, one of high intensity (H trial: 8 cows, -64% of dry matter intake) and one of moderate intensity (M trial: 8 cows, -20% of dry matter intake). Milk sampled before and after 5 days of these restriction periods was used to explore its miRNome.

## Results

### Milk miRNome description

#### Characterization of milk miRNomes

**Fig. 1:**
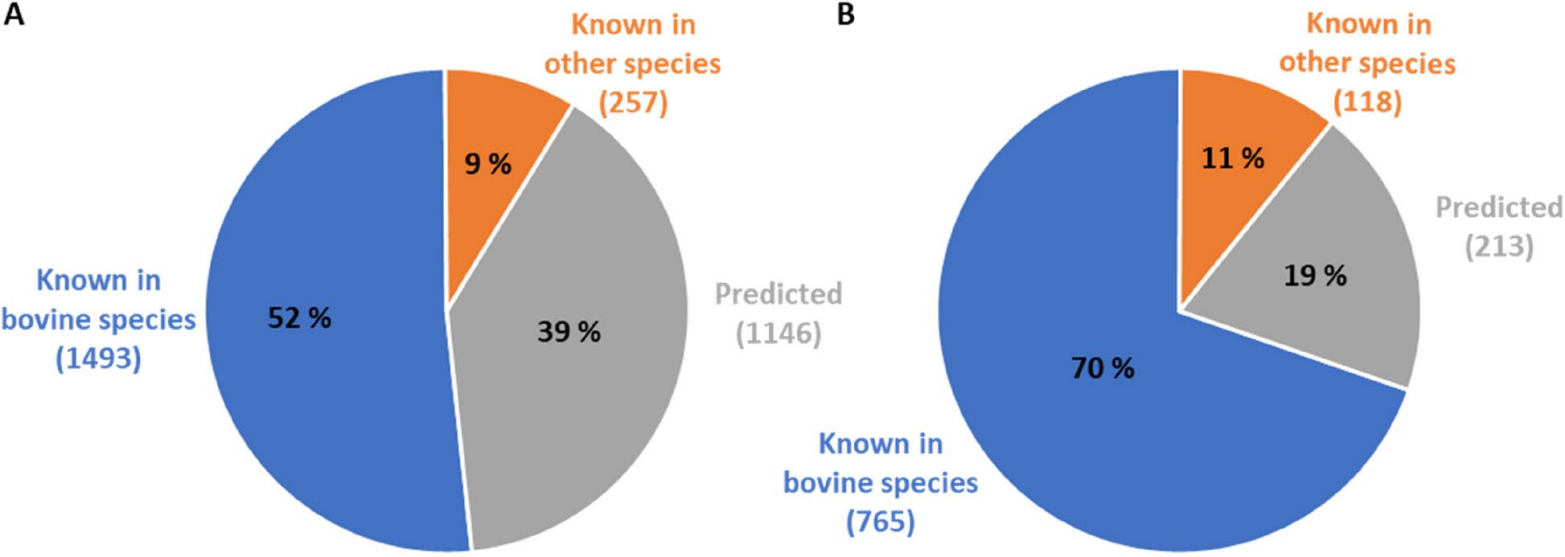
Description of the milk miRNome, all fractions included, without (**A**) and with (**B**) filtering sequencing data according to the HTSfilter (35 read threshold). Proportions (%) and number of predicted and annotated microRNAs in all milk fractions from 16 Holstein cows in two trials, according to their annotation in the miRBase (v.22) and RumimiR (v. Jan2020) databases. **A** All 2,896 identified sequences. **B** All 1,096 filtered sequences with a maximal count across all individuals higher than 35

MiRNA sequencing was performed on samples collected before and during the feed restriction periods, from 3 different milk fractions per trial, and with 8 cows in both trials. All samples combined, an average of 21,679,931 raw reads was obtained per cow, ranging from 266,868 to 57,668,292. After library adaptor removal, size filtering and alignment against the BosTau8 bovine genome, an average of 6,357,421 reads was obtained, ranging from 16,864 to 18,546,736 (Table 1). Analysis of these mapped sequences using miRDeep2 enabled the identification of 2,896 mature miRNA in total, among which 1,493 were already known in the bovine species, 257 in other species and 1,146 were predicted according to miRBase (v. 22) and RumimiR (v. Jan2020) identifications (Fig. 1). Among miRNA known in other species, 27 species were used, with human (hsa), mouse (mmu), goat (chi), rat (rno), sheep (oar), and rhesus macaque (mml) being the most frequently used, in decreasing order. A threshold of 35 reads was identified using the HTSFilter package [18] to maximize filtering similarity among the samples, leaving 1,096 informative miRNA whose maximal count across all individuals was higher than 35, including 765 known in bovine species and 118 in other species.

**Table 1:** Sequencing data regarding microRNAs present in whole milk, fat globules, extracellular vesicles and mammary epithelial cells of bovine milk. Average raw reads (a) and processed reads (b), after the removal of library adapters, size filtering and BosTau8 genome mapping, are described (means with SEM) for trials of high (H) and moderate (M) intensity, before and during feeding restriction

	H trial				M trial			
	Before restriction		During restriction		Before restriction		During restriction	
	Raw reads ^a^	Cleaned and mapped reads^b^	Raw reads	Cleaned and mapped reads	Raw reads	Cleaned and mapped reads	Raw reads	Cleaned and mapped reads
Milk	19,668,749	5,577,054	27,378,695	7,060,167	19,065,157	1,385,673	15,213,021	3,255,062
	± 2,882,695	± 758,482	± 4,675,234	± 1,173,512	± 1,659,929	± 479,980	± 3,248,374	± 868,807
Fat globule	17,146,767	2,939,260	29,444,670	6,319,271	22,955,351	9,740,397	22,170,334	8,898,399
	± 3,382,333	± 643,065	± 5,643,853	± 1,077,091	± 3,805,403	± 1,875,462	± 4,046,845	± 1,649,146
Extracellular vesicles	20,937,675	13,223,662	21,931,929	11,161,224				
	± 1,085,448	± 346,432	± 1,392,881	± 487,097				
Mammary epithelial cells					21,297,121	3,174,956	22,949,700	3,553,923
					± 2,875,821	± 313,792	± 1,862,078	± 335,483

^a^average raw reads

^b^average processed reads

**Fig. 2:**
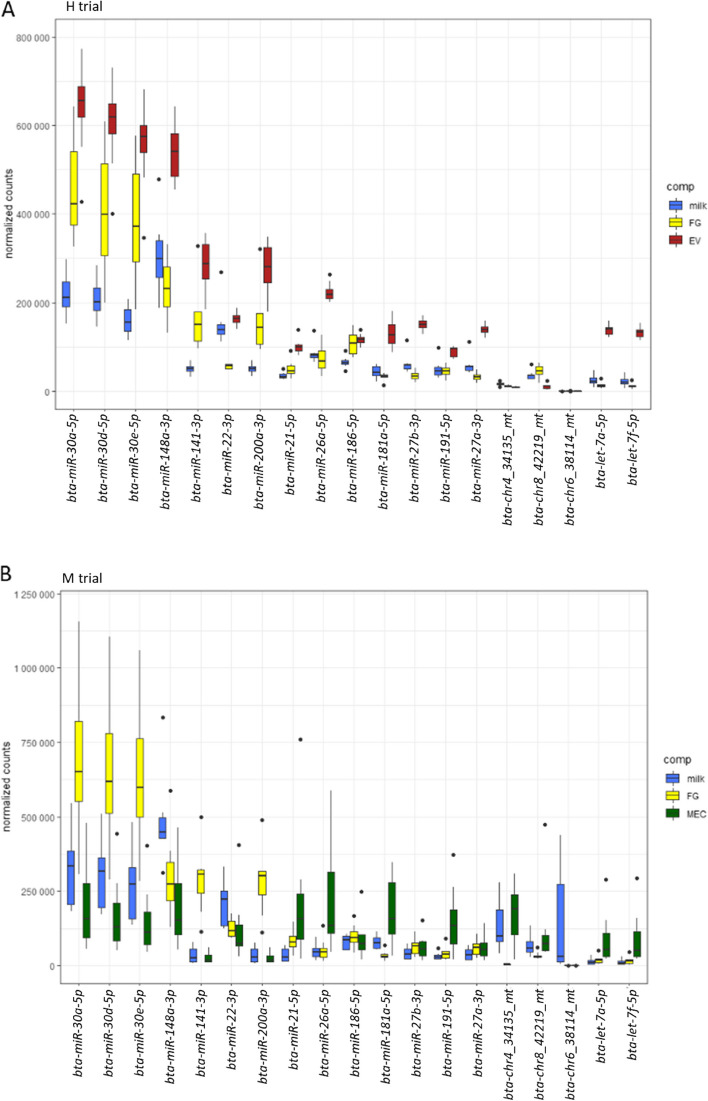
Major milk microRNAs with a mean abundancy of more than 10,000 RPM in any milk fraction during the standard feeding period. **A** Samples of whole milk (milk), milk fat globules (FG) and milk extracellular vesicles (EV) from 8 cows in the high intensity trial (H). **B** Samples of whole milk, milk fat globules and mammary epithelial cells (MEC) exfoliated in milk from 8 cows in the moderate intensity trial (M)

During the standard feeding period, in both trials, four miRNA (*bta-miR-30a-5p*, *bta-miR-30d-5p*, *bta-miR-30e-5p*, and *bta-miR-148a-3p*) were far more abundant than others, in all the fractions combined, representing 41% of all reads. There were 19 miRNA with a read abundance higher than 10,000 reads per million (RPM), representing 76% of all RPM (Fig. 2).

Both trials presented fairly similar miRNome with only 6 miRNA exclusive to the H trial (*bta-chr5_36803_mt*, *bta-chr29_29952_mt*, bta-chr5_37043_mt, bta-chr17_13618_mt, bta-chr21_21449_mt, and *bta-chr13_7384_mt*) and 8 exclusive to the M trial (*bta-chr10_3293_mt*, bta-chr18_15104_st, *bta-chr29_29819_st*, *bta-chr22_23508_mt*, *bta-chr16_12527_mt*, *bta-chr13_7806_mt*, *bta-chr19_17032_mt*, and *bta-chr17_13437_mt*), all of them being predicted and of low abundance. But more miRNA displayed variations in abundance between the trials during standard feeding. In whole milk the abundance of 201 miRNA varied between trials (*P* < 0.05), including 3 (*bta-miR-181a-5p*, *bta-chr4_34135_mt*, and *bta-chr6_38114_mt*) of the 19 most abundant miRNA, while in fat globules (FG) the abundance of 66 miRNA varied between trials (*P* < 0.05), including 2 (*bta-miR-22-3p* and *bta-chr4_34135_mt*) of the 19 most abundant miRNA. 25 miRNA displayed variations in abundance between trials in both whole milk and FG.

#### miRNome variations among milk fractions

To evaluate miRNome variations among different milk fractions, the miRNome of whole milk, FG, extracellular vesicles (EV; only for H diet) and MEC (only for M diet) were compared during the pre-restriction period under standard feeding. The total number of miRNA identified during this period, with a maximal count across all individuals higher than 35, was 1,095.

**Fig. 3:**
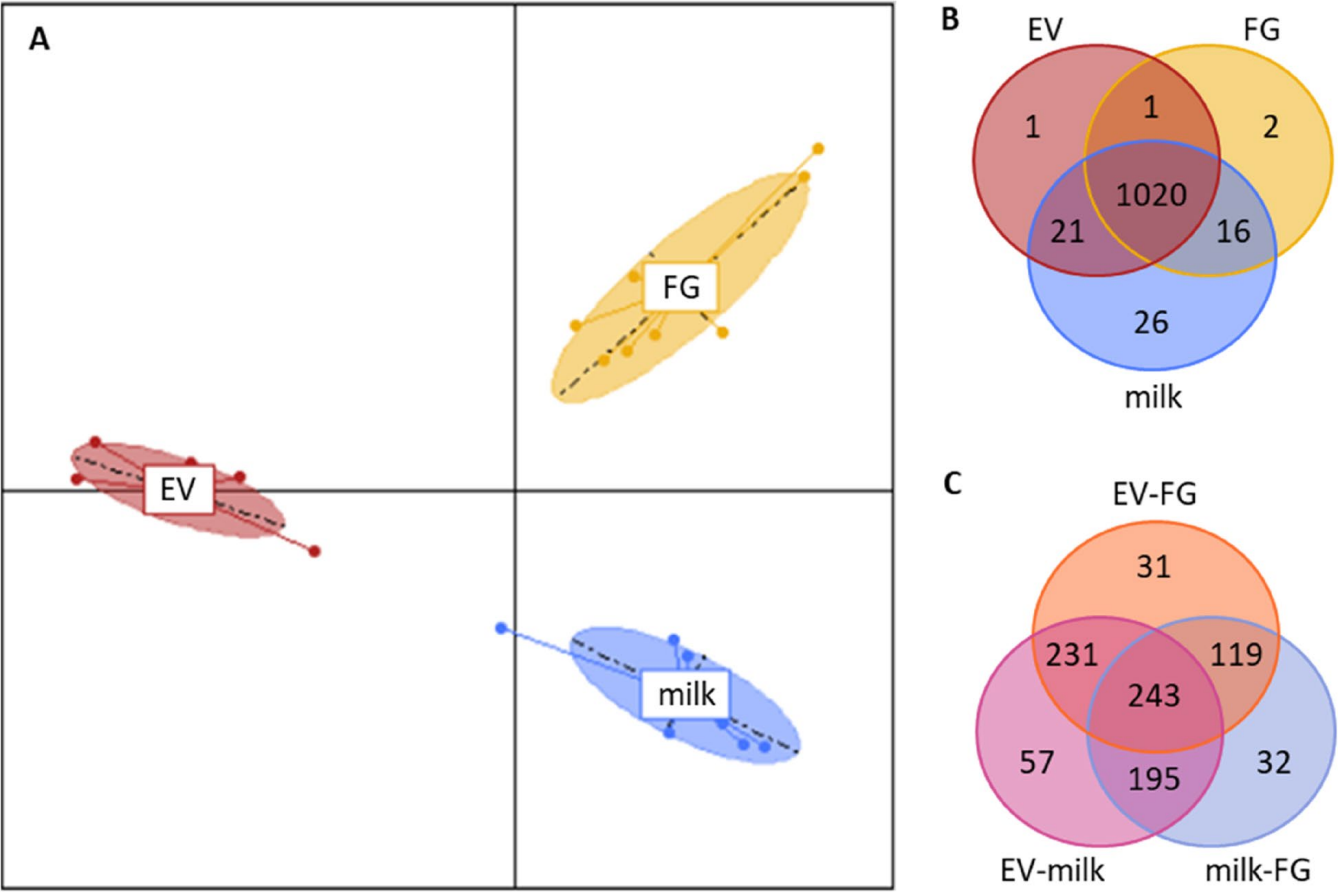
Comparison of milk fraction miRNome from 8 Holstein cows during the standard feeding period of the high intensity trial (H). **A** Principal component analysis with individuals plotted according to their coordinates on the first two components and inertia ellipses, where 95% of individuals are likely to lie within, characterizing the dispersion of each fraction. **B** Venn diagram showing microRNAs present in milk fat globules (FG), milk extracellular vesicles (EV) and whole milk (milk). **C** Venn diagram showing microRNAs whose abundance in milk varied between pairs of fractions: EV and FG, EV and milk, and milk and FG

In H samples, the miRNome differed significantly among milk fractions (Fig. 3A), even in the top 10 most enriched sequences (Table 2). 1,020 miRNA were present in all fractions, with only two miRNA exclusive to FG (*bta-chr4_34864_mt* and *bta-miR-12000-3p*), one to EV (*bta-chr29_30565_mt*) and 26 to whole milk (Fig. 3B). However, differential analysis revealed that 908 miRNA presented variable abundances among fractions, with 624 varying between EV and FG, 726 between EV and milk and 589 between FG and milk (*P* < 0.05; Fig. 3C; Supplementary data S1).

**Table 2:**
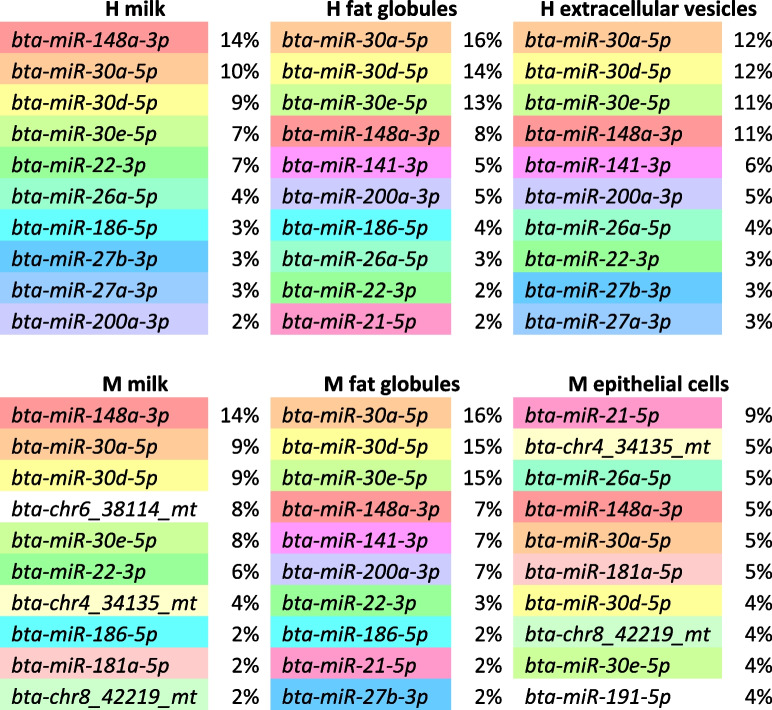
The ten most enriched miRNA in each milk fraction and their relative abundance in percentages (%) according to the total miRNA reads from the high intensity (H) and moderate intensity (M) trials. One color is assigned to each miRNA

**Fig. 4:**
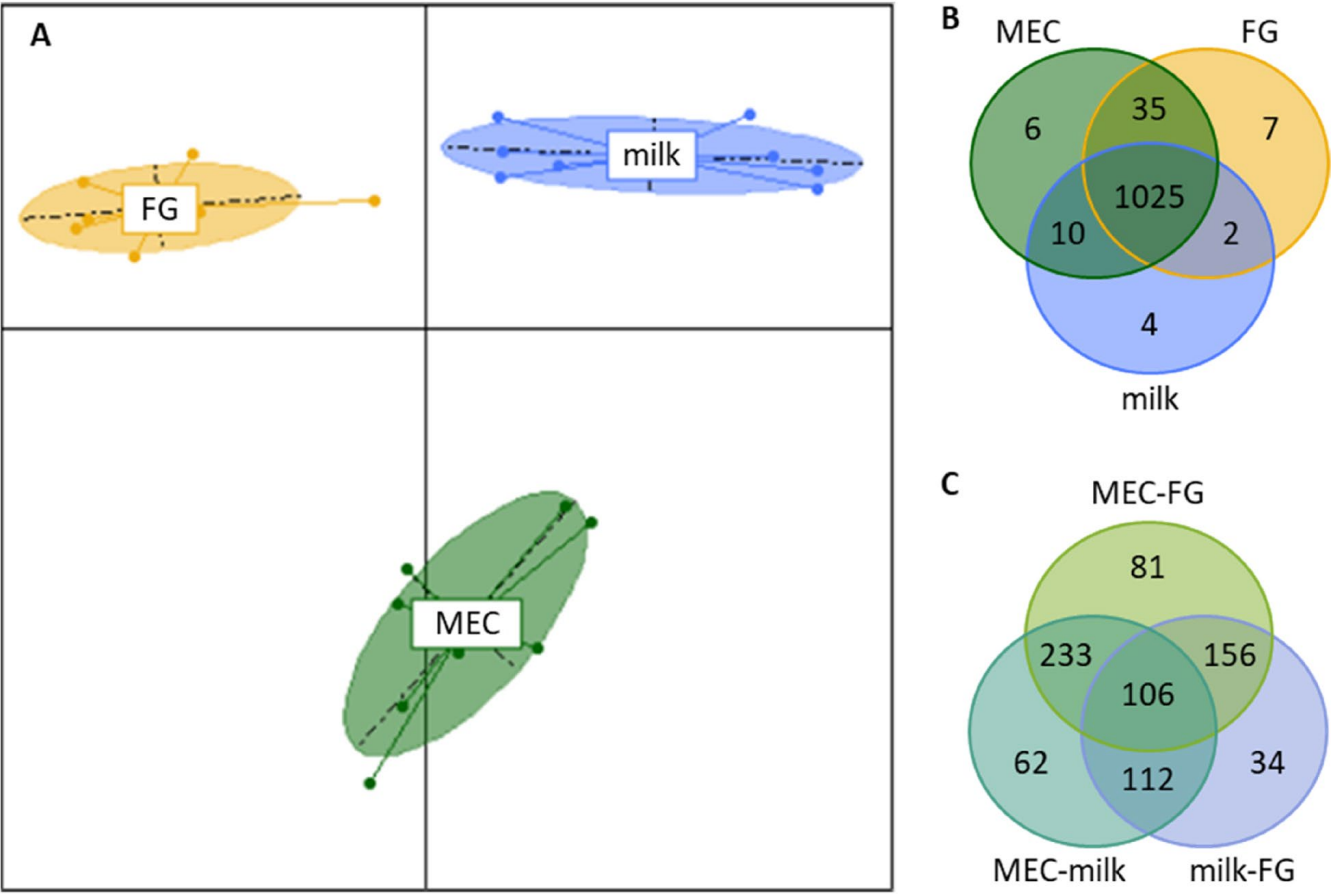
Comparison of the miRNome of milk fractions from 8 Holstein cows during the standard feeding period of the moderate intensity trial (M). **A** Principal component analysis with individuals plotted according to their coordinates on the first two components and inertia ellipses, where 95% of individuals are likely to lie within, characterizing the dispersion of each fraction. **B** Venn diagram showing microRNAs present in milk fat globules (FG), mammary epithelial cells exfoliated in milk (MEC) and whole milk (milk). **C** Venn diagram showing microRNAs whose abundance in milk varied between pairs of fractions: MEC and FG, MEC and milk, and milk and FG

In M samples, the miRNome differed significantly among milk fractions (Fig. 4A) even in the top 10 most enriched sequences (Table 2). 1,025 miRNA were present in all fractions, 7 were exclusive to FG (*bta-chr10_3293_mt*, *bta-chr22_23146_mt*, *bta-chr1_580_mt*, *bta-chr5_35639_mt*, *bta-chr29_29795_mt*, *bta-chr16_12527_mt*, and *bta-chr19_16187_mt*), 6 to MEC exfoliated in milk (*bta-chr18_15104_mt*, *bta-chr23_24155_mt*, *bta-chr29_30565_mt*, *bta-chr18_15104_st*, *ssc-miR-155-3p*, and *bta-chr17_13437_mt*) and 4 to whole milk (*bta-chrX_45480_mt*, *bta-chr25_26942_mt*, *bta-chr13_7806_mt*, and *bta-chr19_17032_mt*) (Fig. 4B). However, differential analysis revealed that 784 miRNA presented variable abundances among fractions, with 576 varying between MEC and FG, 513 between MEC and milk and 408 between FG and milk (*P* < 0.05; Fig. 4C; Supplementary data S1).

Nine hundred ninety four miRNA were common to all samples from both trials, representing 91% of all filtered miRNA. Among them, the 19 major milk miRNA were all featured. Among miRNA exclusively in whole milk, just two (*bta-chrX_45480_mt* and *bta-chr25_26942_mt*) were common to both trials and present at a very low abundance (fewer than 1 of mean RPM) except for *bta-chrX_45480_mt* in M whole milk samples (179 RPM). None were present exclusively in FG in both trials. Among the miRNA whose abundance varied between FG and whole milk fractions, four were common to both trials: *bta-miR-2284a-5p*, *bta-miR-2284f-5p*, *bta-miR-2284g-5p*, and *bta-miR-2285bh-5p*. 36 miRNA had different degrees of abundance between all four fractions in both trials, including some abundant miRNA that are well known in the bovine species: *bta-miR-181a-5p* which is one of the 19 miRNA with more than 10,000 RPM on average in all milk fractions combined and essentially abundant in the MEC fraction (Table 2); *bta-miR-26b-5p*, *bta-miR-192-5p*, and *bta-miR-215-5p* that presented more than 1,000 RPM on average; and *bta-miR-223-3p*, *bta-miR-486-5p*, and *bta-miR-142-3p* that presented more than 100 RPM on average. The abundance profiles of these miRNA may have been characteristic of each milk fraction.

### Impact of feed restriction on milk miRNomes

In the H trial, 1,087 miRNA were present in whole milk, 1,072 in FG and 1,059 in EV. Among them, 4, 3 and 9 were exclusively present during the pre-restriction period and 4, 33 and 16 were exclusively present during the restriction period in whole milk, FG and EV, respectively. All miRNA exclusively observed under one feed condition presented a very low number of reads, with the mean RPM ranging from 0.01 to 2.3 under the conditions in which they were observed.

**Fig. 5:**
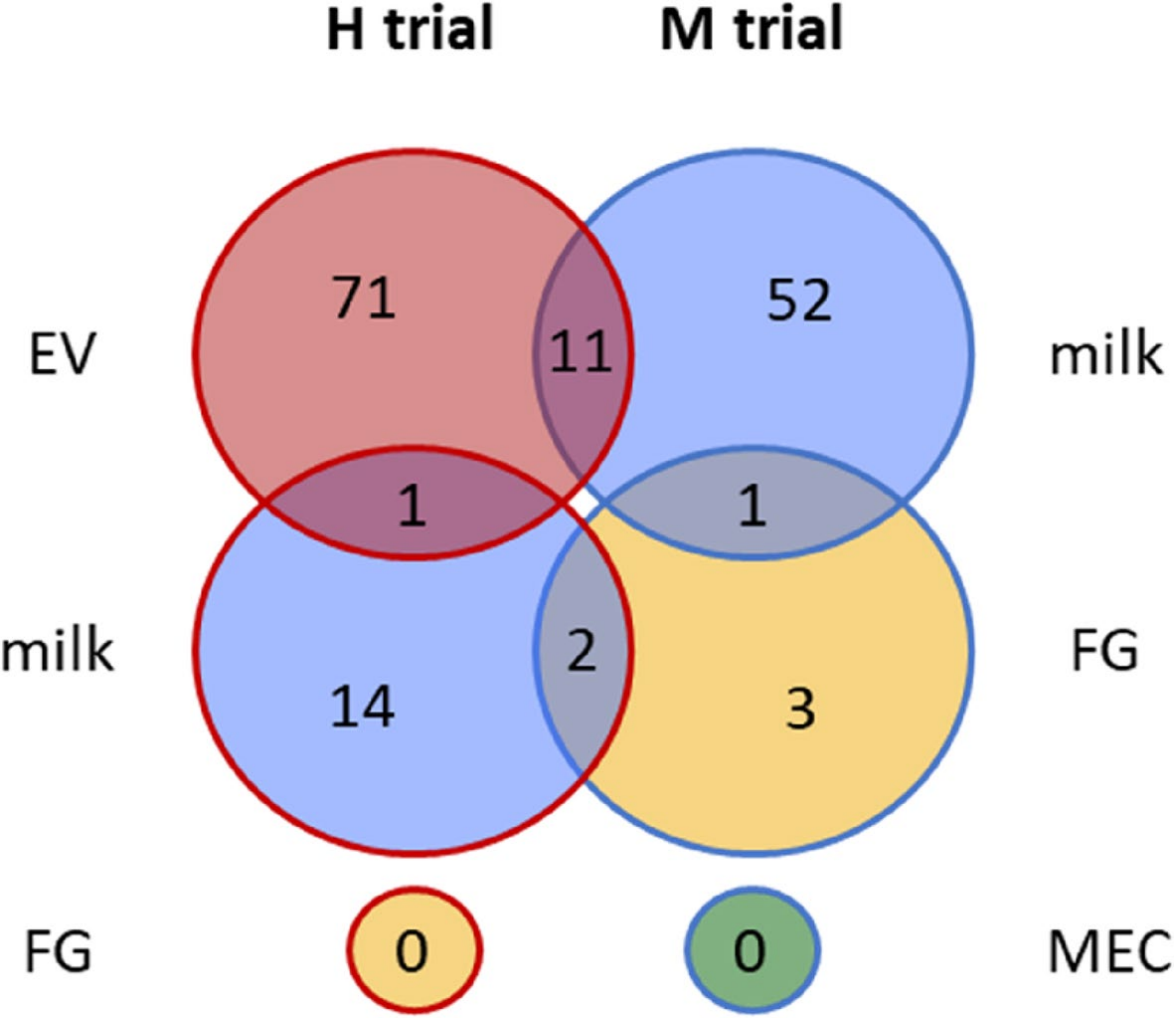
Venn diagram representing microRNAs whose abundance in milk varied in each fraction during feed restrictions: extracellular vesicles (EV), whole milk (milk) and fat globules (FG) in the high intensity restriction trial (H); and milk, FG and mammary epithelial cells (MEC) in the moderate intensity restriction trial (M)

**Fig. 6:**
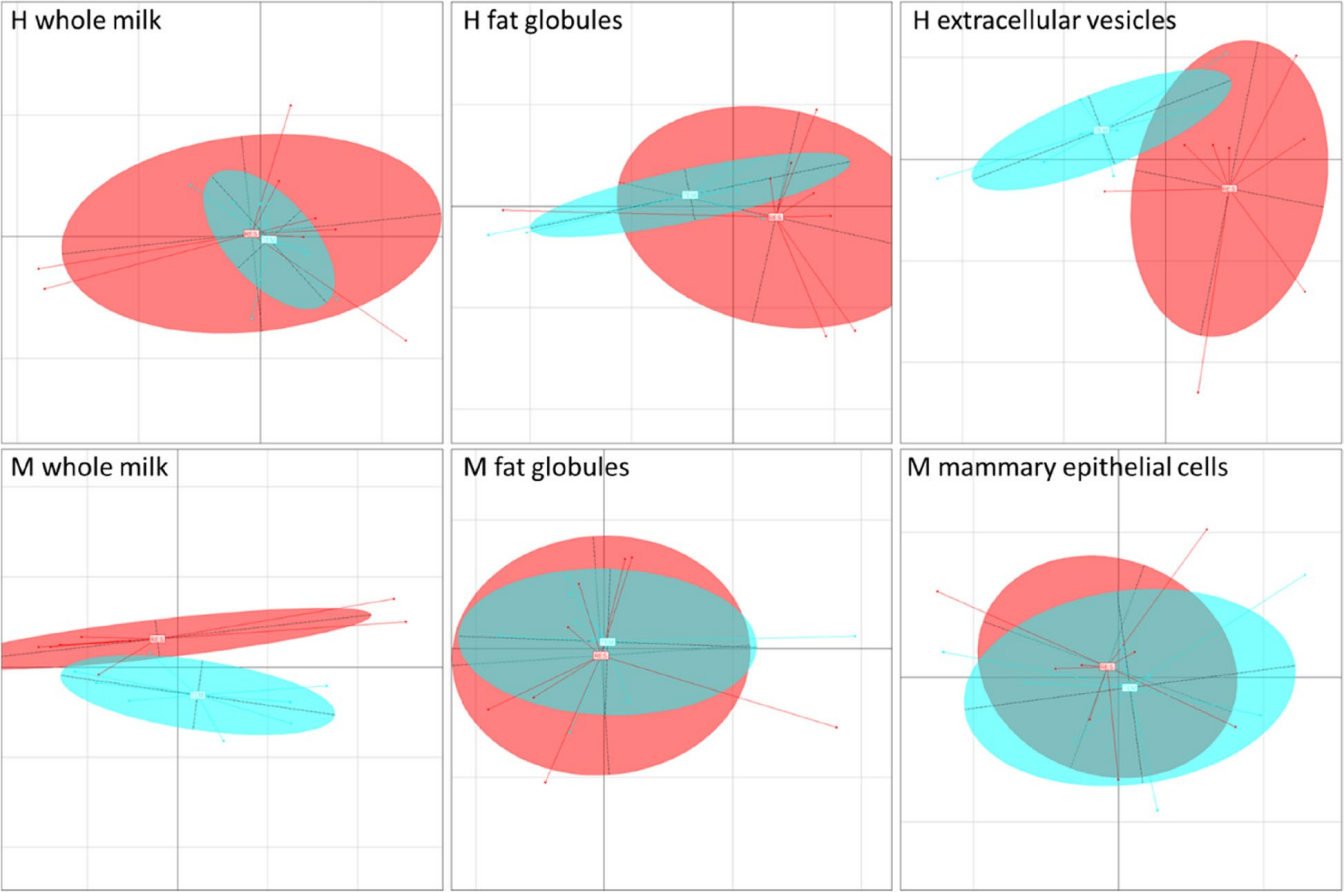
Effect of feed restriction on the milk fraction miRNome after 5 days of high intensity (H trial: 8 cows, -64% of dry matter intake) or moderate intensity (M trial: 8 cows, -20% of dry matter intake) feed restriction trials. Principal component analysis with individuals plotted according to their coordinates on the first two components, and inertia ellipses where 95% of individuals are likely to lie within, characterizing the dispersion within each feed condition: before restriction in cyan and during restriction in red

Ninety-nine miRNA showed significant variations in abundance during H feed restriction: 17 of them displayed significant differences in whole milk samples and 83 in EV samples (one of them being common). No difference was observed in the FG sample (Fig. 5). Among these 99 miRNA with variable abundance, 60 were more abundant during feed restriction than during the pre-restriction period and 39 were less abundant (Supplementary Data S2). Of the 17 miRNA with variations due to feed restriction in whole milk, the abundancy of five of them ranged between 100 and 1,000 RPM (*bta-miR-429-3p*; *bta-chr14_9640_mt*; *bta-chr3_33057_mt*; *bta-miR-486-5p*; and *bta-miR-326-3p*) and none were over 1,000 RPM. Of the 83 miRNA with variable abundance in EV, five displayed abundance between 1,000 and 10,000 RPM (*bta-miR-26b-5p*; *bta-miR-200c-3p*; *bta-let-7g-5p*; *bta-miR-192-5p*; and *bta-miR-25-3p*) and the abundance of 12 was between 100 and 1,000 RPM. Only *bta-chr3_33057_mt* varied in both whole milk and EV fractions, with decreased abundances during feed restriction periods. According to these analyses, EV corresponded to the fraction presenting the highest miRNA variations during the H trial (Fig. 6).

In the M trial, 1,076 miRNA were detected in whole milk, 1,082 in FG and 1,083 in MEC. Of these, 9, 16 and 4 were exclusively identified during the pre-restriction period and 35, 13 and 7 exclusively present during the restriction period in whole milk, FG and MEC, respectively. All miRNA that were exclusively present during one feed condition displayed very low read numbers, with the mean RPM ranging from 0.01 to 3.0 under the condition where they were found, except for *bta-chr6_38143_mt* with 80 RPM on average during pre-restriction in whole milk.

Of the 69 miRNA displaying significant variations in abundance during the M feed restriction, 64 were localized in whole milk and 6 in FG (one of them being common). No differences were observed in the MEC sample (Fig. 5). Among these 69 differently abundant miRNA, 10 were more abundant during feed restriction and 59 were more abundant during the pre-restriction period (Supplementary Data S2). Of the 64 miRNA in whole milk with variable abundance during feed restriction, that of 11 of them ranged from 1,000 to 10,000 RPM (*bta-chr6_38114_mt*; *bta-chr20_20763_mt*; *bta-miR-423-5p*; *bta-chr4_34135_st*; *bta-chr21_22422_mt*; bta-chr12_6503_mt; bta-chr17_13327_mt; *bta-chr17_14009_mt*; *bta-chr3_32262_mt*; *bta-chr8_42857_mt*; and *bta-chr5_35699_mt*) and that of 23 ranged from 100 to 1,000 RPM. Of the 6 miRNA with variable abundance in FG, only *bta-miR-486-5p* had an abundance of between 100 and 1,000 RPM. *Bta-miR-451-5p* was the only miRNA to vary in both whole milk and FG fractions, with increased abundances during feed restriction. According to these analyses, whole milk is the fraction presenting the greatest miRNA variations during the M trial (Fig. 6).

The comparison of the experiments revealed that 13 miRNA were significantly affected by both feed restriction experiments. In the whole milk of the H trial and FG of the M trial, *bta-miR-486-5p* presented increased abundance and *bta-chr21_21585_mt* decreased abundance during feed restriction. The 11 others varied in the H trial (EV) and M trial (whole milk fractions): *bta-miR-223-3p* and *bta-miR-142-5p* increased in abundance during feed restriction; *bta-chr20_21167_mt*, *bta-chr15_10516_mt*, *bta-chrX_45078_mt*, *bta-chr20_20849_mt*, *bta-chr6_38228_mt*, and *bta-chr17_13703_mt* decreased in abundance during feed restriction; *hsa-miR-532-3p*, *bta-chr2_19057_mt*, and *bta-chr8_42857_mt* increased in abundance in EV of the H trial while abundance decreased in the whole milk fraction of the M trial. No miRNA displayed significant variations in abundance in both trials whole milk nor FG fractions (Fig. 5).

### Functional pathways potentially affected by feed restriction

The Tarbase v8 and Diana mirPath v3 bioinformatics tools were used to explore the pathways affected by the 17 known miRNA whose abundance was higher than 100 RPM and varied significantly in response to feed restriction: nine miRNA identified during the H trial (one in whole milk and eight in milk EV) and ten during the M trial (nine in whole milk and one in milk FG) (Table 3). Target predictions for these 17 miRNA led to the identification of 41 significantly enriched pathways, involving 7 to 17 miRNA that target 6 to 140 genes in these pathways, so a total of 1,378 genes potentially affected (Table 4, Supplementary Data S3). Among these, some key pathways for lactation were highlighted, such as the regulation of fatty acid and milk fat metabolism, the regulation of protein processing and the regulation of cell cycle and apoptosis, as well as pathways involved in stress responses such as the immune response, epithelial membrane integrity and hypoxia.

**Table 3:** List of the 17 miRNA displaying abundancies higher than 100 RPM (reads per million) and significant variations according to feed restriction in different milk fractions (milk, whole milk; FG, fat globules; EV, Extracellular Vesicles) analyzed for target prediction functional analysis using the Tarbase v8 and Diana mirPath v3 bioinformatics tools

	Trial	Fraction	Mean RPM	Adj. *p*-value	Fold change
*bta-miR-486-5p*	H	milk	287	0.034	4.43
	M	FG	285	0.003	7.09
*bta-miR-26b-5p*	H	EV	4,910	0.021	1.16
*bta-miR-200c-3p*	H	EV	1,851	0.037	1.32
*bta-let-7 g-5p*	H	EV	1,796	0.011	1.19
*bta-miR-192-5p*	H	EV	1,518	0.034	1.21
*bta-miR-25-3p*	H	EV	1,340	0.007	1.33
*mml-miR-106b-3p*	H	EV	163	0.014	1.21
*bta-miR-223-3p*	H	EV	132	0.003	18.14
*bta-miR-142-5p*	H	EV	387	0.007	9.14
	M	milk	255	0.003	5.03
*bta-miR-423-5p*	M	milk	8,866	0.001	0.29
*bta-miR-423-3p*	M	milk	956	0.007	0.30
*bta-miR-125a-5p*	M	milk	940	0.036	0.45
*efu-miR-30a-3p*	M	milk	652	0.035	2.46
*bta-miR-125b-5p*	M	milk	371	0.036	0.49
*bta-miR-181a-2-3p*	M	milk	171	0.014	0.29
*bta-miR-92b-3p*	M	milk	143	0.011	0.27
*mmu-miR-200a-5p*	M	milk	138	0.042	2.59

**Table 4:** Significantly enriched functional union pathways of genes targeted by known microRNAs with an abundancy of more than 100 RPM in one milk fraction and significantly affected by feed restriction, from Tarbase v8 and Diana mirPath v3 with a *P*-value threshold of 0.05. ^*^ *p*-value resulting of DIANA miRPath analysis showing the probability that the examined pathway is significantly enriched with gene targets of the selected miRNAs

KEGG pathway	*p*-value*	Number of genes	Number of miRNA
Protein processing in endoplasmic reticulum	8E-11	115	16
Hippo signaling pathway	2E-07	91	16
Cell cycle	9E-07	83	16
p53 signaling pathway	1E-06	53	16
Fatty acid metabolism	2E-06	28	16
Lysine degradation	2E-06	30	15
Adherens junction	2E-05	52	16
TGF-beta signaling pathway	2E-05	54	16
Other types of O-glycan biosynthesis	3E-05	18	13
Glycosaminoglycan biosynthesis—chondroitin sulfate / dermatan sulfate	4E-05	13	10
N-Glycan biosynthesis	1E-04	32	14
Spliceosome	1E-04	86	16
Endocytosis	2E-04	120	17
Sphingolipid signaling pathway	2E-04	71	17
Thyroid hormone signaling pathway	2E-04	72	17
Inositol phosphate metabolism	3E-04	41	15
HIF-1 signaling pathway	3E-04	68	16
TNF signaling pathway	3E-04	69	17
FoxO signaling pathway	6E-04	85	17
Phosphatidylinositol signaling system	0.001	50	16
Fatty acid biosynthesis	0.002	6	10
Ubiquitin mediated proteolysis	0.002	85	17
MAPK signaling pathway	0.004	140	17
Proteasome	0.004	31	13
ErbB signaling pathway	0.005	53	16
Glycosaminoglycan biosynthesis—keratan sulfate	0.005	9	10
Oocyte meiosis	0.005	65	16
DNA replication	0.006	23	11
Signaling pathways regulating pluripotency of stem cells	0.007	80	16
Steroid biosynthesis	0.007	13	7
Focal adhesion	0.007	116	17
RNA transport	0.008	94	17
RNA degradation	0.011	50	16
Estrogen signaling pathway	0.011	56	16
Neurotrophin signaling pathway	0.014	69	17
B cell receptor signaling pathway	0.017	43	17
mTOR signaling pathway	0.018	38	16
Wnt signaling pathway	0.020	76	16
ECM-receptor interaction	0.021	40	15
mRNA surveillance pathway	0.023	55	16
Biosynthesis of unsaturated fatty acids	0.029	13	15

## Discussion

During this study, a total of 2,896 mature miRNA were characterized in milk bovine fractions, including 1,493 that were already known in the bovine species. First, compared to available miRNome descriptions in the literature, the number of miRNA identified was slightly higher but of the same order of magnitude as previously reported in whole milk by Le Guillou et al*.* [4], who identified 2,038 mature miRNA in Holstein milk, including 900 annotated in the bovine species. Among the 19 major milk miRNA whose average abundancy was higher than 10,000 RPM during standard feeding, 13 were also found with more than 10,000 RPM on the Holstein milk miRNome as described by Le Guillou et al*.* [4], including the top 10 of them. Nine of the top 10 miRNA observed in bovine milk exosomes by Yun et al*.* [19] and seven of the top 10 miRNA observed by Cai et al*.* [20] in bovine milk EV were also present, with more than 10,000 RPM, in our EV fraction. Six of the top 10 miRNA observed in non-pasteurized cow milk fat by Golan-Gerstl et al*.* [21] were also in our top 10 miRNA present in FG during standard feeding. Among the 18 most expressed miRNA in the bovine MEC line MAC-T cells [22], 11 were also present with more than 10,000 RPM in exfoliated MEC during our study. Li et al*.* [8] also compared the miRNome of bovine milk fractions, and among the miRNA they observed with more than 10,000 RPM, 9 out of 18 in fat, 12 out of 20 in somatic cells and 9 out of 16 in whey were also higher than 10,000 RPM in fat, MEC and whole milk, respectively, in our study. Similarly, Benmoussa et al*.* [12] compared the top 10 most enriched miRNA sequences in different milk fractions and species across several studies, and observed that the same most abundant miRNA were found recurrently across studies. They only recorded 27 different miRNA reaching this top 10 abundance in all bovine studies, all milk fractions combined, including eight of the top 10 miRNA present in each milk fraction during our study, thus supporting its accuracy. The current study also described new highly abundant miRNA in milk that had not been previously reported, including some that were predicted and not yet included in the miRbase (v.22) and RumimiR (v. Jan2020) databases (*chr4_34135_mt*, *chr6_38114_mt*, and *chr8_42219_mt*).

The different milk fractions all presented a very similar miRNA composition, with 994 out of 1,095 miRNA being common to all fractions across both restriction trials. This agreed with the findings of Li et al*.* [8] who observed high similarities between milk fat and whey (87% shared miRNA) but to a lesser extent with milk somatic cells (75% shared miRNA). These authors hypothesized that the differences with milk somatic cells were due to their heterogeneity, as they consist of both immune and exfoliated epithelial cells. In our study, exfoliated MEC were purified using immunomagnetic separation to prevent such heterogeneity. This purification led to greater miRNome similarities between milk MEC and other milk fractions than those previously observed with somatic cells. Nevertheless, although the miRNA composition was quite similar, their relative abundances varied between fractions, which finally enabled the discrimination of these different fractions. When looking at top 10 miRNA in each fraction, these differences were noticeable, particularly for *miR-148a-3p* which is the most abundant miRNA in the milk of numerous species, including ruminants and humans [12]. Although it was the most abundant miRNA in whole milk of both trials, *miR-148a-3p* was not the most abundant in FG, EV or MEC during our study. Differences in abundance between milk fractions, even within the most abundant miRNA, had also been observed in previous studies [8, 21]. This attested that each milk fraction has its own miRNome, the specificity of which is determined by the relative abundance of each miRNA.

When comparing both feed restriction trials, their differences in lactation day should be reminded, as M trial started at 77 ± 5 days in milk and H trial started at 165 ± 21 days in milk. While being distant in time, both occurred during mid-lactation, in its declining phase. Moreover, in milk FG, it has been shown that variation of miRNome occurred more between lactogenesis, galactopoiesis and involution than within the same lactation phase [23]. Indeed, between day 70 and day 170 of lactation, they only reported one differentially expressed miRNA.

Feed restriction significantly affected the milk miRNome, with 102 miRNA exclusive to one feeding condition in at least one milk fraction, and 155 miRNA whose abundance varied during the feed restriction. All exclusive miRNA presented fewer than 3 RPM on average, except *bta-chr6_38143_mt* with 80 RPM, and may be of little interest as biomarkers as their detection in milk may not be reproducible. As for the miRNA whose abundance varied with the feed restriction, only *bta-chr6_38114_mt* was part of the most abundant miRNA in milk (more than 10,000 RPM on average), and yet this miRNA was only found to be very abundant in whole milk during the M trial. Nevertheless, 53 miRNA with an abundance of more than 100 RPM on average varied during feed restriction and could be of interest in terms of discriminating energy balance status. Some of these are known to directly affect lactation metabolism. The key role in lactation regulation of *miR-486-5p*, which was more abundant in whole milk samples of the H trial (Fold change (FC) = 4.43; *P* = 0.03) and in FG samples of the M trial (FC = 7.09; *P* = 0,003) during the feed restriction period than during the standard feeding period, has been described as notably being involved in increasing the secretion of triglycerides, β-casein and lactose [24]. This higher abundance of *miR-486-5p* was not concordant with the lower de novo synthesis of fatty acids observed during the H feed restriction [25], the lower β-casein production observed during both the H and M restrictions [26] and the lower lactose content observed during the M feed restriction [27]. This increase in *miR-486-5p* could result from a concentration effect due to the lower milk volume or its greater secretion from the mammary epithelium to milk during the feed restriction period than during the standard feeding period. The expression of *miR-26b-5p*, which was more abundant in EV samples from the H trial (FC = 1.16; *P* = 0.02) during feed restriction could be related to the composition of milk fatty acids, as described in the mammary cells of goats [28] which was also concordant with the decrease in de novo fatty acid synthesis recorded during the H feed restriction [25]. The abundance of *bta-miR-25-3p* increased in EV during the H trial (FC = 1.33; *P* = 0.007); its role in the inhibition of triacylglycerol synthesis and lipid accumulation has been reported in a previous study performed on goat mammary epithelial cells [29]. Differences in the expression of *bta-miR-25-3p* in the mammary tissue between cow breeds with different dairy performances and negative energy balance susceptibility have also been described [30]. Milk fat metabolism may also be promoted by *miR-142-5p* in goat MEC [31], whose abundance increased in both whole milk samples from the M trial (FC = 5.03;* P* = 0.003) and EV samples from the H trial (FC = 9.14;* P* = 0.007) during feed restriction periods. The abundance of *bta-let-7g-5p* increased in EV during H feed restriction (FC = 1.19;* P* = 0.01); the inhibition role of this miRNA on β-casein protein synthesis and MEC differentiation has also been reported in a mouse model [32]. Finally, *miR-200c-3p*, whose abundance increased in EV during H feed restriction (FC = 1.32; *P* = 0.04), is a member of the *miR-200* family and is thus known for its effect on mammary gland morphogenesis [33]. Its lower abundance during feed restriction was consistent with the reorganization of mammary gland tissue (which could lead to mammary gland involution) that has been observed during an intense feed restriction experiment [34].

The milk miRNome was not affected similarly in the different milk fractions. No significant miRNA variations were observed within the CEM fraction and only six miRNA varied in FG during feed restrictions. As such, miRNome variations seemed to be linked mainly to variations in miRNA abundance in the skimmed fraction of milk and milk EV. EV was the fraction presenting the greatest number of miRNA affected by feed restriction, with 83 variable miRNA. Benmoussa et al. [10] suggested that most milk miRNA is encapsulated within EV, which agrees with the protection against degradation provided by this encapsulation [35]. EV incorporate miRNA specifically and are involved in intercellular communication, so that their miRNome may thus indicate the nature and physiological status of the cells from which they derive [36]. During this study, no specific EV populations were purified, and the miRNome observed was a combination of miRNA derived from milk exosomes, microvesicles and apoptotic bodies. These milk EV, mainly derived from MEC and immune cells [37], should thus reflect the adaptation of these cells to the negative energy balance induced by feed restriction. In the current study, this was explored through the prediction of variable miRNA targets and an in silico study of the metabolic pathways in which they are involved.

An in silico functional analysis was performed to identify the targeted genes and the known miRNA with variations in abundance due to feed restriction and their metabolic pathways. Among the genes directly targeted by these miRNA, 14 corresponded to proteins found to be variable in milk during the same experiments [26]. In fact, during the H trial, GAPDH, ALDOA, ACTN4, and PGK1 proteins were only present in milk during the feed restriction period; ENO1, FN1, ACTB, ACTG1, and EEF1A1 proteins were significantly more abundant and HSPA8, RAP1B, CD36, ARF1 and SAR1A proteins were significantly less abundant in milk during the feed restriction period. Moreover, during the M trial, alpha-enolase, encoded by *ENO1*, was also found to be more abundant in milk during the feed restriction period. Known miRNA with variations in abundance during feed restriction targeted some key pathways for lactation such as the regulation of fatty acid and milk fat metabolism, the regulation of protein processing and the regulation of cell cycle and apoptosis, as well as pathways involved in stress responses, including the immune response, epithelial membrane integrity and hypoxia. The regulation of fatty acid metabolism had also been shown by Billa et al*.* [25] during the H trial with a decreased milk content in short chain fatty acids (< C16) during restriction due to reduced de novo synthesis, and an increased content in long chain fatty acid (> C16), suggesting increased mobilization from adipose tissue. This regulation of fatty acid metabolism was also consistent with the increased plasma concentrations of non-esterified fatty acids observed in both the M and H trials [25, 27]. The regulation of protein metabolism has previously been detailed for both the M and H trials [26] and was in accordance with the miRNA epigenetic regulation predicted in this study. The miRNA affected by the feed restriction conditions that we studied here were also involved in protein synthesis (targeted pathways related to protein processing in the endoplasmic reticulum and glycan biosynthesis) and also in protein degradation, with pathways related to ubiquitin mediated proteolysis and proteasome. Some proteins are also involved in immune system regulation and may be under the regulation of miRNA, as shown by the targeted genes involved in the TNF signaling pathway and B cell receptor signaling pathway. Another targeted pathway concerned the adherens junction, which is essential for integrity of the mammary epithelial barrier. Herve et al*.* [27] described an increased MEC exfoliation rate during the M feed restriction period; these MEC had lost their connection with the epithelium, which could have been linked to a loss of mammary epithelium integrity which we also showed in the M trial through an increase in milk Na^+^.

When comparing feed restrictions of different intensities, the H feed restriction led to more variations in milk miRNA than the M feed restriction, suggesting a greater modification of gene regulation during a more marked negative energy balance. This is in line with the proteomic variations observed in milk during the same trials, where more variations to milk protein abundance were observed during the H feed restriction than during the M feed restriction [26].

## Conclusions

This study explored the bovine milk miRNome under feed restrictions and compared these effects on different milk fractions, and indeed, although 90% of milk miRNA were observed in all milk fractions, the abundance of 1,027 miRNA varied between the fractions, revealing a specific miRNome for each milk fraction. Feed restrictions exerted different effects on the miRNome of each milk fraction. In fact, the 155 miRNA whose abundance varied during feed restriction were mainly located in whole milk and EV, whereas FG and exfoliated MEC were little or not affected. Moreover, more miRNome variations appeared to occur when the restriction was more intense. In silico analysis of functional pathways targeted by variable miRNA under feed restriction reflected modifications to certain key pathways for lactation related to milk fat and protein metabolism, cell cycle and stress response. These findings open opportunities for future research on the use of milk miRNA as biomarkers of energy status in dairy cows.

## Methods

### Animals, experimental designs, and sampling

This study included the results of two feed restriction trials: one of high intensity (H) and the other of moderate intensity (M).

The H trial was conducted at the INRAE Herbipôle experimental farm (UE Herbipôle, 15,190 Marcenat, France; https://doi.org/10.15454/1.5572318050509348E12). All procedures involving animals were approved by the local Ethics Committee for the Auvergne-Rhône-Alpes region and the French Ministry of Higher Education, Research and Innovation (APAFIS #3737–2015043014541577v2).

Eight multiparous mid-lactation (165 ± 21 days in milk; lactation ranks 2 to 5) Holstein cows were used to study the effects of six days of feed restriction designed to meet 50% of their net energy for lactation (NE_L_) requirements, as described by Billa et al*.* [25]. During the pre-restriction period, the cows were fed ad libitum with a total mixed ration. During the restriction period, the feed allowance was reduced by 64% to meet 50% of individual NE_L_ requirements calculated from body weight, dry matter intake and milk yield and composition, as recorded during the pre-restriction period. The milk samples used in this study were collected during morning milking, before feed distribution, at days -2 and 5 relative to the initiation of feed restriction.

The M trial was performed at the INRAE PEGASE experimental farm (IE PL, 35,650 Le Rheu, France; https://doi.org/10.15454/yk9q-pf68). All procedures involving animals were approved by the local Ethics Committee in Animal Experimentation for Rennes and the French Ministry of Higher Education, Research and Innovation (APAFIS #3063–2015110215066393).

Eight primiparous or multiparous peak lactation (77 ± 5 days in milk; lactation ranks 1 to 4) Holstein cows were used to study the effects of 29 days of feed restriction designed to reduce their dry matter intake by 20%, as described by Herve et al*.* [27]. During the pre-restriction period the cows were allowed an ad libitum intake of a total mixed ration. During the restriction period the cows were fed 80% of their ad libitum dry matter intake, as recorded during the pre-restriction period. The milk samples used in this study were collected during morning milking, before feed distribution, at days -7 and 5 relative to the initiation of feed restriction.

### Sample preparation

#### Milk fat globule collection

Milk FG were isolated from residual milk samples as described by Pawlowski et al*.* [38]. The milk samples were centrifuged immediately at 2,000 g for 10 min at 4°C to isolate fat. One g of the fat supernatant layer was then placed in 2.0 mL TRIzol LS solution (Invitrogen Life Technologies Inc., Carlsbad, CA) and stored at − 80°C.

#### Mammary epithelial cell purification from milk

MEC were purified from fresh milk (1.8 kg) using an immunomagnetic separation technique as described by Herve et al*.* [39]. The purified milk MEC suspension was stored at -80°C in 1 mL TRIzol (Invitrogen Life Technologies) until RNA extraction was performed.

#### Extracellular vesicle isolation

The isolation of milk-derived EV, as well as their validation and exo-RNA isolation, were performed by Excilone (Elancourt, France) as previously reported [40]. Skimmed milk samples were obtained by centrifuging 50 mL whole milk at 3,000 g for 30 min at 4°C (Allegra X-15R, Beckman Coulter, France). The whey part of milk was obtained after acid precipitation with 10% (v/v) 10% acetic acid, incubation at 37°C for 10 min and 10% (v/v) 1M sodium acetate for 10 min at room temperature followed by centrifugation at 1,500g, 4°C for 15 min and filtration using the vacuum-driven filtration system Millipore Steritop, 0.22 μm. The whey supernatants were concentrated using Amicon 100kDa centrifugal filter units (Merck Millipore, Burlington, MA) at 4,000 g and 20°C up to a final volume of 6 mL. The obtained retentate was ultra-centrifuged to pellet the EV at 100,000 g for 1h10 at 4°C (Beckman Coulter, Optima XPN-80, 50TI 155 rotor). The pellets were solubilized in 500 μL PBS then loaded onto 11 mL of pre-prepared sucrose gradient 5–45% and ultra-centrifuged at 200,000 g for 18h at 4°C (Beckman Coulter, Optima XPN-80, SW41 rotor). Fractions of 1 mL were collected and the selected fractions containing the targeted exosome population (fractions 10–12) were diluted in 6 mL PBS 1X and finally centrifuged at 100,000 g for 1h10 at 4°C (Beckman Coulter, Optima XPN-80, 50TI rotor). The pellets were resuspended in 50 μL PBS 1X and then pooled and stored at -80°C until further analyses.

### RNA isolation

Total RNA, including small RNAs, were isolated from 500 µL whole milk using the RNA NOW kit (Ozyme, Saint-Cyr-l'Ecole, France), with overnight precipitation to guarantee a maximum yield of small RNA. The concentration and integrity of the RNA were assessed by spectrophotometry (Nanodrop, ND-1000). The RNA samples were stored at -80°C until required for further processing.

Similarly, total RNA from milk FG and MEC were isolated using the TRIzol LS and TRIzol (Invitrogen Life Technologies) protocols, respectively, with overnight precipitation to guarantee a maximum yield of small RNA. The RNA were dissolved in 10 µL RNase-free water for MEC isolations and 50 μL RNase-free water for other milk fractions, and their concentrations were quantified by ND-1000 NanoDrop™ Spectrophotometer. The RNA were then stored at -80 °C until use.

The isolation of total RNA from EV samples was performed using an optimized mirVana Total RNA Isolation Kit (Invitrogen Life Technologies) with some modifications: first, Trizol LS Reagent (Ambion) was used for initial cell disruption instead of mirVana Phenol Lysis Reagent, followed by the addition of chloroform, ethanol precipitation (sample:100% ethanol ratio of 1:1.25, v/v) and mirVana kit column fractionation. Glycogen was added to improve RNA recovery. To obtain high quality RNA, a DNAse I treatment (Qiagen, Hilden, Germany) was performed on the columns for 15 min at RT according to the manufacturer’s instructions. Finally, the RNA were eluted in 50 µL Elution buffer. The RNAs were quantified using a Bioanalyzer 2100 instrument (Agilent, Santa Clara, CA), Pico chip and ND-1000 NanoDrop™ Spectrophotometer (Ozyme).

### Small library preparation and sequencing

Small RNA libraries were prepared using the Illumina TruSeq Small RNA Library Prep Kit (Illumina, San Diego, CA) with RNA isolated from the different milk fractions, representing a total of 96 samples. This was performed according to the manufacturer’s instructions, with PCR amplification up to 13 cycles, by the GenomEast Platform (IGMBC, Illkirch, France).

The single-read sequencing of libraries was carried out on six lanes on an Illumina HiSeq 4000 sequencer by the GenomEast Platform (IGMBC). RNA sequencing data were subsequently deposited in the Gene Expression Omnibus (GEO): GSE207759.

### Data analysis

Raw sequences were cleaned from adapters and filtered for size (17–28 nt) with Cutadapt [41]. Cleaned sequences were clustered into unique reads and mapped to the bovine reference genome bosTau8 using the mapper.pl module from the miRDeep2 software [42]. Novel miRNA and precursors were identified using the miRDeep2 core module miRDeep2.pl. Novel miRNA datasets were created by adding miRNA predicted with a miRDeep2 score > 0 to known miRNA (miRBase v.22 [43]). Quantification was done using the quantifier.pl miRDeep2 module, and the quantification results were filtered with a custom Perl script parse_miRDeep2_outputs.pl (https://forgemia.inra.fr/‌sylvain.marthey/paqmir/blob/master/paqmir_postprocess_quantifier/parse_miRDeep2_output.pl) to eliminate any redundancy between known and predicted novel miRNA. Mature miRNA known in other species or predicted unknown were searched for in the RumimiR database (v. Jan2020) [44] in order to identify ruminant miRNA already described in the literature but not listed in the outdated last version of miRBase (v.22).

### Statistical analysis

Statistical analyses were performed using R software v3.6.3 (R Development Core Team, 2020, http://www.R-project.org). The filtering method from the HTSFilter package [18] was used to remove miRNA that appeared to generate an uninformative signal. Tests for differential expression were only applied to miRNA whose maximal count across all four samples was higher than the threshold found with HTSFilter. Principal component analyses were performed using the ade4 package v1.7.15 [45], followed by pair-wise differential analyses between the miRNomes of milk sampled before and during feed restriction with the DESeq2 package v3.11 [46]. MiRNome variations were firstly explored between milk fractions during the standard feeding period, and then between standard and restricted feeding periods within each fraction.

### Target prediction

The Tarbase v8 [47] and Diana mirPath v3 [48] software programs were used to predict genes targeted by known miRNA with at least 100 reads per million mapped reads (RPM) in one milk fraction and to identify metabolic pathways affected by the regulation of these targeted genes. Default options in miRPath were used, the pathways union option and FDR correction have been applied for the pathway analysis, and the enrichment analysis method used the Fisher’s exact test with a p-value threshold of 0.05.

### Supplementary Information

**Additional file 1. **

**Additional file 2. **

**Additional file 3. **

## Data Availability

The miRNomic data are available on the Gene Expression Omnibus (GEO) data repository with the dataset identifier GSE207759.

## Abbreviations

EV: Extracellular vesicles
FC: Fold change
FG: Fat globules
H: High intensity
M: Moderate intensity
MEC: Mammary epithelial cells
miRNA: MicroRNA
NE_L_: Net energy for lactation
RPM: Reads per million mapped reads
